# *MECP2* mRNA Profile in Brain Tissues from a Rett Syndrome Patient and Three Human Controls: Mutated Allele Preferential Transcription and In Situ RNA Mapping

**DOI:** 10.3390/biom15050687

**Published:** 2025-05-08

**Authors:** Martina Mietto, Silvia Montanari, Maria Sofia Falzarano, Elisa Manzati, Paola Rimessi, Marina Fabris, Rita Selvatici, Francesca Gualandi, Marcella Neri, Fernanda Fortunato, Miryam Rosa Stella Foti, Stefania Bigoni, Marco Gessi, Marcella Vacca, Silvia Torelli, Joussef Hayek, Alessandra Ferlini

**Affiliations:** 1 Medical Genetics Unit, Department of Medical Sciences, University of Ferrara, 44121 Ferrara, Italy; mttmtn@unife.it (M.M.);; 2 Pathology Institute, Fondazione Policlinico Universitario A. Gemelli IRCCS, 00168 Rome, Italy; 3 Institute of Genetics and Biophysics “A. Buzzati-Traverso”, CNR, 80131 Naples, Italy; 4 The Dubowitz Neuromuscular Centre, UCL Great Ormond Street Institute of Child Health, London WC1E 6BT, UK; 5 Child Neuropsychiatry Unit, University Hospital, Azienda Ospedaliera Universitaria Senese, 53100 Siena, Italy

**Keywords:** Rett syndrome (RTT), *MECP2* transcription profiling, human brain, X-chromosome inactivation (XCI)

## Abstract

Rett syndrome (RTT) is a rare X-linked dominant neurodevelopmental disorder caused by pathogenic variants in the methyl-CpG-binding protein 2 (*MECP2*) gene, which encodes a methyl-CpG-binding protein (MeCP2) that acts as a repressor of gene expression, crucial in neurons. Dysfunction of MeCP2 due to its pathogenic variants explains the clinical features of RTT. Here, we performed histological and RNA analyses on a post-mortem brain sample from an RTT patient carrying the p.Arg106Trp missense mutation. This patient is part of a cohort of 56 genetically and clinically characterized RTT patients, for whom we provide an overview of the mutation landscape. In the RTT brain specimen, RT-PCR analysis detected preferential transcription of the mutated mRNA. X-inactivation studies revealed a skewed X-chromosome inactivation ratio (95:5), supporting the transcriptional findings. We also mapped the *MECP2* transcript in control human brain regions (temporal cortex and cerebellum) using the RNAscope assay, confirming its high expression. This study reports the *MECP2* transcript representation in a post-mortem RTT brain and, for the first time, the in situ *MECP2* transcript localization in a human control brain, offering insights into how specific *MECP2* mutations may differentially impact neuronal functions. We suggest these findings are crucial for developing RNA-based therapies for Rett syndrome.

## 1. Introduction

Rett syndrome (RTT, OMIM 312750) is a rare, X-linked dominant neurodevelopmental disorder caused by monoallelic pathogenic variants in the methyl-CpG-binding protein 2 gene (*MECP2,* OMIM 300005). Predominantly affecting females, RTT has an estimated prevalence of 1 in 10,000 to 15,000 live births [1] and is one of the most common genetic causes of intellectual disability (ID) in females. The clinical phenotype is characterized by an initial period of normal development, followed by regression in language and motor skills, particularly hand function, and progressive neurological deterioration [2]. The *MECP2* gene, located on Xq28, consists of four exons coding for the methyl-CpG-binding protein 2 (MeCP2). This gene is highly susceptible to a variety of mutation types, including missense, nonsense, frameshift, and copy number variations, such as deletions and duplications. Notably, eight “hotspot” variants account for more than 70% of RTT cases, most of which involve C-to-T transitions at CpG methylated dinucleotides. These sites are prone to spontaneous deamination, leading to thymine conversion and subsequent mutations.

The *MECP2* gene encodes two main splicing isoforms, which differ in their exon composition, translational start sites, and resulting protein molecular weights. The longer and predominant isoform, expressed in the central nervous system (CNS), has its ATG in exon 1, which splices to exons 3 and 4 (*MECP2_e1*), producing a protein of 498 amino acids (aa). The second shorter isoform, with the ATG in exon 2, splices to exons 3 and 4 (*MECP2_e2*), yielding a protein of 486 aa. Despite their predicted molecular weights of approximately 50 kDa, both isoforms exhibit an apparent electrophoretic mobility around 70 kDa in Western blot experiments, likely due to post-translational modifications. Both isoforms retain the methyl-binding domain (MBD), which is essential for binding to 5-methylcytosine (5mC), the transcriptional repression domain (TRD), which interacts with histone deacetylase and the transcriptional corepressor SIN3A, and the C-terminal domain (CTD) [3].

RTT patients invariably carry monoallelic de novo *MECP2* mutations, while hemizygous male patients often present with a non-specific ID phenotype. *MECP2* duplications represent a distinct clinical variant of RTT known as *MECP2* duplication syndrome, which almost exclusively occurs in males and is characterized by moderate to severe ID. Collectively, *MECP2* mutations give rise to a broad phenotypic spectrum of developmental disorders, including autism, X-linked recessive mental retardation, or severe neonatal encephalopathy in males, as well as dominant RTT clinical variants in females.

MeCP2 is ubiquitously expressed, with the highest levels found in the CNS, where it contributes to the regulation of neuronal development, synaptic function, and neuroplasticity [1,4]. MeCP2 acts as a transcriptional repressor by linking methylated DNA to deacetylated histones. MeCP2 binds to 5mC sites, leading to the recruitment of co-factors that orchestrate transcriptional repression, such as histone deacetylase 3 (HDAC3), transducing beta-like protein 1 (TBL1), nuclear receptor co-repressor-silencing mediators of retinoic acid and thyroid hormone receptor (NCoR-SMRT), and G-protein suppressor 2 (GPS2). MeCP2 binding induces a closed chromatin configuration, which is disrupted in mutated MeCP2 proteins that are unable to recruit these co-repressors, resulting in a lack of transcriptional silencing and an open chromatin configuration. In addition to its role as a transcriptional repressor, MeCP2 also acts as a transcriptional activator via cAMP response element-binding protein (CREB1) and serves as a splicing and chromatin architecture modulator [3,5,6].

The precise mechanism by which de novo, monoallelic *MECP2* mutations severely affect neuronal and astrocyte function by disrupting synaptic connections and leading to RTT phenotype remains unclear, suggesting an etiology based on haploinsufficiency. Haploinsufficiency is widely considered a key contributor, but skewed X-chromosome inactivation (XCI) may also play a significant role. While most studies of XCI patterns in RTT patients have been performed on peripheral blood cells—which may not accurately represent brain XCI status—the XCI profile in the brains of affected individuals has been poorly studied. This gap is particularly significant given our molecular understanding from mouse models, where comprehensive studies of conditionally deleted *Mecp2* in adult hippocampus demonstrate that its loss triggers immediate transcriptome dysregulation, accompanied by genome-wide histone modification changes, including reduced H3 acetylation as early molecular events [7]. Limited studies have characterized *MECP2* expression patterns in human brain tissues, with only a handful of reports documenting transcript levels and protein distribution in both neurotypical controls and RTT patients. Zito and Lee [8] observed remarkable variability in the expression of *MECP2*, *CDKL5*, and *FMR1* in human adult and fetal brains from multiple donors. This variability was evident not only across cell types but also between donors. Their transcription analysis revealed that *MECP2* was primarily transcribed in the fetal brain’s cortex, while in the adult brain, it was predominantly expressed in the cortex, cerebellum, and basal nuclei. This study also found that MeCP2 is expressed in neurons, granule cells, and glia, highlighting its widespread role in brain function [8].

Fetit et al. [9] investigated MeCP2 protein expression in post-mortem brain tissue from a patient with RTT and autism spectrum disorders (ASDs). They reported that while the cortical layer structure remained intact, there were reductions in neuron numbers and aberrant myelination, suggesting underlying neuropathological changes [9].

Finally, Pejhan et al. [10] conducted immunohistochemical analyses of RTT and control brains, revealing lower MeCP2 expression in glial cells of the white matter. Interestingly, neurons did not show a significant reduction in MeCP2 levels compared to control brain tissue, underscoring the cell-type-specific effects of *MECP2* mutations [10].

In this study, we analyzed a cohort of 56 individuals, all carrying pathogenic *MECP2* variants. Our investigation aimed to characterize genetic and clinical diversity within this cohort, providing insights into the frequency and distribution of different *MECP2* mutations. Innovatively, we performed *MECP2* genetic analysis, RT-PCR profiling, and XCI studies on a post-mortem brain specimen from an RTT patient carrying a de novo p.Arg106Trp (R106W) missense mutation, which is known to be associated with a severe RTT phenotype. The RTT patient passed away at 11 years of age. Our results show a skewed XCI pattern that positively selects the mutated allele, leading to preferential transcription of the mutated mRNA. We also demonstrated that the mutation is heterozygous in brain DNA. To further characterize the spatial localization of the *MECP2* transcript, we applied RNAscope in situ hybridization (ISH) assay to control brain regions, specifically in the temporal cortex and cerebellum. Our findings support a brain-confined loss-of-function pathogenesis for RTT and confirm high MeCP2 expression in the brain, along with a cell-type-specific mapping. By integrating genetic, molecular, and histological analyses, our study contributes to a more comprehensive understanding of RTT pathogenesis and reinforces the significance of MeCP2 dysfunction in the disease’s clinical presentation.

## 2. Materials and Methods

All methods were carried out in accordance with relevant guidelines and regulations.

### 2.1. Patient Cohort

Our cohort consisted of 54 female and 2 male patients with a clinical phenotype compatible with RTT. Patients were classified into classic and atypical RTT according to international criteria [11]. All cases underwent *MECP2* molecular analysis at the Laboratory of Medical Genetics Unit of Ferrara University Hospital from May 2000 to May 2024 and resulted positive for likely pathogenic (LP)/pathogenic (P) variants. Informed consent was obtained from all subjects enrolled in this study.

### 2.2. MECP2 Sequence Analysis

Genomic DNA was extracted from EDTA-preserved whole blood using the BioRobot Universal System and the QIAsymphony Instrument and Kits, following the manufacturer’s instructions (QIAGEN, Aarhus, Denmark). All exons, flanking intron regions, and the 5′ UTR and 3′ UTR regions of the *MECP2* gene were sequenced in all 54 females by Sanger sequencing using the BigDye Terminator Cycle Sequencing Kit, according to the manufacturer’s instructions (Applied Biosystems by Thermo Fisher Scientific, Waltham, MA, USA), on the ABI Prism 3130XL automated sequencer (Applied Biosystems). Primers and PCR amplification conditions have been previously reported [12].

### 2.3. MECP2 Deletion and Duplication Analysis

Multiplex ligation probe amplification (MLPA) assay was performed on all female patients who tested negative by Sanger sequencing and the 2 male patients, using the P015 *MECP2* Kit according to the manufacturer’s instructions (MRC Holland, Amstedam, The Netherlands). The reaction products were analyzed using a DNA analyzer (ABI Prism 3130XL, Applied Biosystems), and data analysis was carried out using Coffalyser software v.140721.1958-140701.0000 (MRC Holland). When the MLPA result indicated a single exon deletion or duplication, the result was confirmed using *MECP2* exon-specific Real-time PCR.

### 2.4. Collection of Human Brain Specimens

We had access to a post-mortem brain specimen (temporal cortex) from a girl with RTT (number [N] = 1), carrying the missense mutation p.Arg106Trp, referred by the Child Neuropsychiatry Unit at the University General Hospital (Siena, Italy). This specimen was donated by the parents for scientific purposes, following their signed consent. The girl was born in 1987 and died in 1998 due to a very severe RTT phenotype with respiratory complications.

Control brain samples were obtained from non-oncological neurosurgical procedures at the Histopathology Unit of Policlinico Gemelli Hospital (Rome, Italy) under Ethical Committee approval (see details in “Istitutional Review Board Statement” paragraph).

Before experimental use, the samples were anonymized and then re-evaluated by an expert neuropathologist for quality control assessment to exclude the presence of any additional pathological processes. Human non-RTT (referred to as “control” for brevity) tissues consisted of formalin-fixed-paraffin-embedded (FFPE) sections from a 50-year-old female temporal (CTRL.TC.F) and frontal (CTRL.FC.F) cortex; a 20-year-old male cerebellum (CTRL.CB) and frontal cortex (CTRL.FC), and a 9-year-old male temporal cortex (CTRL.TC).

### 2.5. Histological Characterization of Brain Specimens

Prior to staining, the FFPE sections from both the RTT and control brain (CTRL.TC) temporal cortices were defatted in xylene for 10 min, then hydrated in 100% alcohol for 2 min, followed by rinsing in distilled water. The slices were stained using hematoxylin and eosin (H&E), with hematoxylin staining cell nuclei blue and eosin counterstaining eosinophilic intracellular and extracellular protein structures in shades of red and pink. Images were observed with a Nikon Eclipse 80i fluorescence microscope (Nikon Instruments, Melville, NY, USA) connected to a high-resolution CCD camera (Nikon Instruments). Nis-Elements Viewer software v.5.21.00 (Nikon Systems, Yokohama, Japan) enabled the collection of multiple images, which were stitched together to generate one large image in brightfield at 20× magnification.

### 2.6. X-Chromosome Inactivation Analysis

Genomic DNA was isolated from three FFPE slides of both the RTT and control brain (CTRL.TC.F) temporal cortexes using the QIAamp DNA Tissue Kit according to the manufacturer’s instructions (QIAGEN).

XCI patterns were analyzed using the HUMARA assay. The methylation-sensitive enzyme HpaII digests only the active allele, while MspI, an isoschizomer of HpaII, cuts indiscriminately based on the methylation state, eliminating any amplification product from both the active and inactive alleles [13].

Three reactions were set up: (a) 2 µg of DNA were incubated without the restriction enzymes; (b) 2 µg of DNA was digested with 20 U of HpaII (M-Medical, Milan, Italy), and (c) 2 µg of DNA was digested with 20 U of MspI (M-Medical). All reactions were performed in a total volume of 50 µL and incubated for 16 h at 37 °C. The reactions were then terminated by inactivating the enzymes at 65 °C for 20 min.

Following digestion with the restriction enzymes, 2 µL of each reaction was used in a PCR with a final volume of 50 µL containing 1 µM primers (AR.FW and AR.REV, sequences are provided in Supplementary Table S1), 250 µM dNTPs, 3.5 U ExTaq (Takara Bio, Kusatsu, Japan), and 1× ExTaq buffer. PCR was performed using the following program: 94 °C for 5 min, hold; 94 °C for 45 s, 61 °C for 45 s, 72 °C for 1 min, 35 cycles; 72 °C for 5 min, hold. PCR products were run on 12% polyacrylamide gels and visualized by silver nitrate staining (Supplementary Figure S1).

### 2.7. Gene-Specific and Hot-Stop RT-PCR

Total RNA was extracted from the RTT brain temporal cortex and four control brain tissues (temporal cortex—CTRL.TC, cerebellum—CTRL.CB, and two frontal cortices—CTRL.FC and CTRL.FC.F, Policlinico Gemelli Hospital tissues), as previously described [14].

cDNA synthesis was performed from 1 μg of total RNA. RT-PCR was carried out using a *MECP2*-specific oligonucleotide as the priming oligo (MECP2-long 5′ mapping on exon 2–3 junction; the sequence is provided in Supplementary Table S1).

On the newly synthesized cDNA, RT-PCR was carried out using two primers mapping to exon 3 and exon 4 of the *MECP2* gene, respectively (Rett.FW and Rett. REV, sequences are provided in Supplementary Table S1). The first strand of cDNA was purified by a QIAquick PCR purification kit, according to the manufacturer’s instructions (QIAGEN).

The first strand cDNA was amplified using the following cycling conditions: 94 °C for 1 min, 64 °C for 45 s, 68 °C for 1 min 30 s, 5 cycles; 94 °C for 30 s, 63 °C for 45 s, 68 °C for 2 min, 35 cycles. The PCR products were visualized on a 1% agarose gel by ethidium bromide staining. Then, we blotted the products on a GeneScreen Plus Hybridization Transfer membrane (Revvity, Waltham, MA, USA), hybridized them using an *MECP2* exon 4 probe, and exposed the membrane for at least 4 h for autoradiography.

The RTT brain temporal cortex PCR product was sequenced using the BigDye Terminator Cycle Sequencing Kit according to the manufacturer’s instructions (Applied Biosystems) on the ABI Prism 3130XL automated sequencer (Applied Biosystems).

To precisely quantify the mutated *MECP2* transcript, we carried out a Hot-stop RT-PCR using two primers mapping on exon 3 and exon 4 of the *MECP2* gene (MECP2.3FW and MECP2.4REV, sequences are provided in Supplementary Table S1) to increase amplification stringency. Hot-stop PCR was performed using radioactive labeling with [α-32P]dCTP. As the p.Arg106Trp missense mutation creates a novel additional restriction site within *MECP2* exon 3, the PCR products were then subjected to enzymatic digestion using NlaIII (New England Biolabs, Ipswich, MA, USA). The digestion reaction was carried out for 2 h and 30 min at 37 °C, using 20 units of NlaIII (10 U/µL) in 1× NEBuffer 4, supplemented with 1× BSA. Both the digested and undigested products were resolved on a 12% polyacrylamide gel (acrylamide:bisacrylamide ratio 29:1). Following electrophoresis, the gel was dried at 65 °C for 1 h, then at 72 °C for an additional hour. The dried gel was exposed to autoradiography, and images were acquired using a PhosphorImager (Molecular Dynamics, Sunnyvale, CA, USA). Band quantification was performed using ImageQuant TL 7.0 software with background subtraction (rolling ball algorithm). Automated lane and band detection was followed by manual adjustment to ensure accurate segmentation. Target bands (“a”, “b”, and reference band “d”) were identified, with “d” used for loading normalization.

For the densitometric analysis, the Optical density (OD) values were obtained by integrating pixel intensities within each band region. The relative abundance of band “b” versus “a” was calculated as the ratio of their normalized intensities and expressed as a fold change and a percentage difference.

### 2.8. Western Blot Analysis

Proteins from the RTT brain temporal cortex and two human control brain areas, frontal cortex (CTRL.FC.F) and cerebellum (CTRL.CB) (Policlinico Gemelli Hospital tissues), were extracted in a sample buffer consisting of 75 mM Tris-HCl (pH 6.8), 3.8% SDS, 4 M urea, glycerol, and 2-mercaptoethanol. Soluble proteins (10 μg and 20 μg) were resolved using 6% resolving/4% stacking polyacrylamide gels, followed by electrophoretic transfer to a nitrocellulose membrane (Sartorius, Göttingen, Germany). Nitrocellulose strips were blocked in 5% milk powder in TBST buffer and probed with antibodies against MeCP2 at 1:10 dilution (Santa Cruz Biotechnology, Dallas, TX, USA) and HMG-1 at 1:5 dilution (Santa Cruz Biotechnology). Blots were incubated with the appropriate biotinylated secondary antibodies (Amersham, Buckinghamshire, UK), followed by streptavidin conjugated to horseradish peroxidase (Dako, Glostrup, Denmark). Proteins were visualized using the ECL+ system (Amersham). Bands were detected using the AlphaImager™ Gel Imaging System (Alpha Innotech Corporation, San Leandro, CA, USA), and densitometric analysis was performed using AlphaImager™ IS-2200 software. Band intensities (Integrated Density values) were normalized to HMG1 loading controls, and the mutant/control ratio was calculated from three independent experiments. Statistical significance was assessed by a two-tailed Student’s *t*-test (n = 3 technical replicates per group).

### 2.9. MECP2 RNAscope ISH Analysis in Control Brain Specimens

To evaluate the localization of *MECP2* transcript in human control brain areas (cerebellum—CTRL.CB and temporal cortex—CTRL.TC, Policlinico Gemelli Hospital tissues), we performed an ISH RNAscope assay using RNAscope 2.5 HD Reagent assay Red (Advanced Cell Diagnostics, Newark, CA, USA).

PPIB (Cyclophilin B) was used as the positive control probe, while DapB (4-hydroxy-tetrahydrodipicolinate reductase) was used as the negative probe.

The inventoried probe Hs-MECP2, which targets the *MECP2* transcript, was used. This probe contains 20 Z pairs and targets the nucleotides between 355 and 1417 of NM_001110792.1.

RNAscope assay was performed according to the manufacturer’s instructions with the following optimizations to enhance the specificity of the signal:

- Incubation of FFPE slides for 30 min at 60 °C, after the deparaffinization step;
- Target retrieval step for 10 min;
- Incubation of FFPE slides with Amp 5 for 60 min;
- Protease Plus was used for pre-treatment protease digestion.

All images were observed with a Nikon Eclipse 80i fluorescence microscope (Nikon Instruments) connected to a high-resolution CCD camera (Nikon Instruments). Nis-Elements Viewer software v.5.21.00 (Nikon Systems) enabled the collection of multiple images, which were stitched together to generate one large image in brightfield at 20× magnification.

## 3. Results

### 3.1. MECP2 Mutation Landscape and RTT Clinical Features

**Figure 1:**
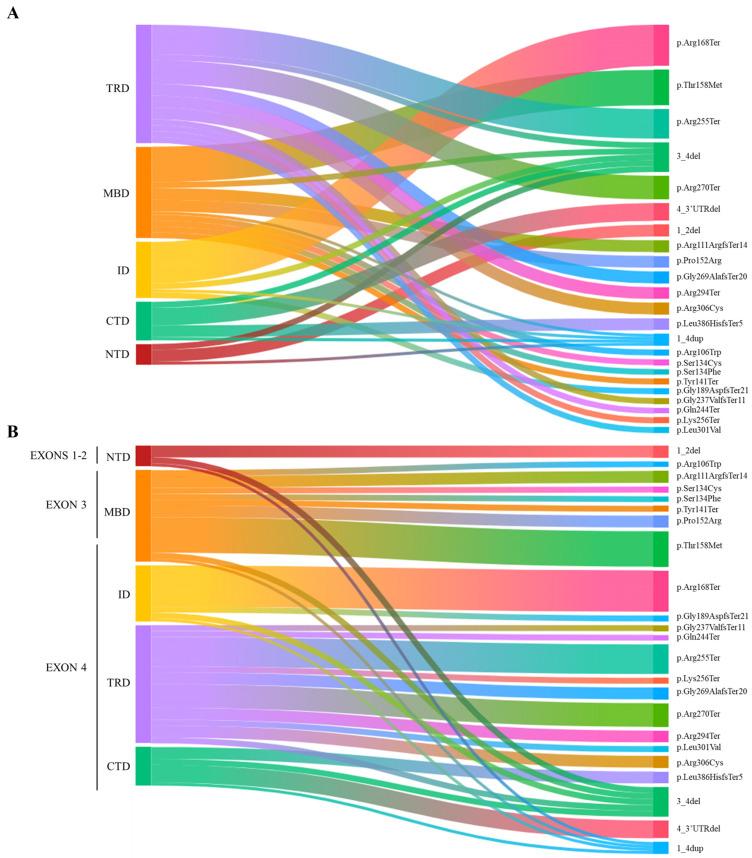
Landscape of *MECP2* pathogenic variants across the different domains and exons of the MeCP2 in our RTT cohort. (**A**) The alluvial plot illustrates the distribution of mutation types depending on their frequency, including 21 nonsense, 15 missense, 9 frameshift mutations, 9 deletions, and 2 duplications across MeCP2 domains. The most common mutations are p.Thr158Met, p.Pro152Arg, p.Arg306Cys (missense mutation), p.Arg168Ter, p.Arg255Ter, and p.Arg270Ter (nonsense mutation). Deletions primarily involve exons 3–4. (**B**) The alluvial plot illustrates the distribution of mutation types based on their 5′-3′ location in the coding sequence. NTD = N-terminal domain; MBD = Methyl-binding domain; ID = Intervening domain; TRD = Transcription repression domain; CTD = C-terminal domain.

Our cohort consisted of 54 females (aged 16 months to 50 years) and 2 males (aged 2 years and 14 years 9 months). All subjects had an identified class IV/V (LP/P) *MECP2* variant. The *MECP2* mutation landscape is shown in Figure 1 (Figure 1A,B).

The mutations identified in female cases can be divided as follows: 15 missense mutations, 9 frameshift mutations, 21 nonsense mutations, and 9 deletions. Among the missense mutations, the most frequent are p.Thr158Met (6 cases), p.Pro152Arg (2 cases), and p.Arg306Cys (2 cases). As for nonsense mutations, the most common are p.Arg168Ter (7 cases), p.Arg255Ter (5 cases), p.Arg270Ter (4 cases), and p.Arg294Ter (2 cases).

Among the deletions, most involved exons 3–4 (four cases). In three cases, the deletion involved exon 4 and its downstream 3′UTR. Exons 1 and 2 were deleted in only two cases. The variations identified in the male cases both involve *MECP2* whole gene duplication.

Our cohort of classical RTT cases has an ethnic distribution primarily of Italian origin, with 38 cases identified. Among these, the two most represented regions are Emilia Romagna, with 17 cases, and Tuscany, with 6 cases. Additionally, six cases originated from foreign countries: three from Hungary, one from Pakistan, one from Ghana, and one from Romania.

The cohort of atypical RTT cases also shows a predominantly Italian ethnic distribution, with eight cases identified. Among these, the two most represented regions are Emilia Romagna, with five cases, and Lombardy, with two cases. In this group, only two cases originated from foreign countries: one from Albania and one from Romania.

The phenotype of the majority of atypical RTT cases is characterized by late regression or no regression at all (which means that the first period of normal development was absent, and psychomotor delay was present from the first months of life). Their global clinical picture was indeed milder compared to classical RTT. One case, which carries the deletion of exons 1–2, presents a severe phenotype that could be described as congenital RTT, characterized by severe cognitive impairment, absence of speech, inability to ambulate, drug-resistant epilepsy, and corpus callosum agenesis.

An additional observation in the RTT cohort concerns the age at diagnosis. When considering 18 years of age as the cutoff for adulthood, we found that 18 cases (16 of whom were classical RTT) out of the total 54 were diagnosed after reaching the age of 18. Among these patients, the mutations identified were distributed as follows (in order of frequency): six cases with frameshift mutations, six cases with nonsense mutations, and four patients with missense variants, specifically p.Pro152Arg, p.Ser134Cys, and p.Thr158Met. Additionally, two patients presented with N-terminal deletions.

On the other hand, 35 cases (27 of whom were classical RTT) out of the total 54 were diagnosed before the age of 18. The mutations identified in these patients were as follows (in order of frequency): 15 cases with nonsense mutations, 11 patients with missense variants, 6 patients with exons 3–4 and 4-3′UTR deletions, and 3 cases with frameshift mutations. For one classical RTT patient, the age at diagnosis is not known.

**Figure 2:**
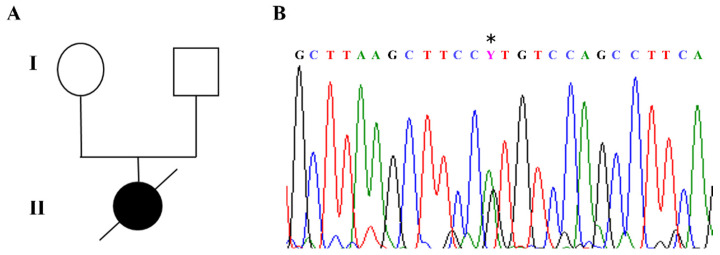
RTT patient index case carrying a de novo p.Arg106Trp missense mutation. (**A**) Pedigree of RTT girl born in 1987 and died in 1998 due to a very severe RTT phenotype with respiratory complications. (**B**) The patient carried a de novo c. 316C>T (*) mutation in exon 3 (reverse strand) of the *MECP2* gene corresponding to p.Arg106Trp (R106W). This pathogenic variant is known to be associated with severe phenotype.

The index case in our cohort carried a de novo c.316C>T heterozygous pathogenic variant in exon 3 of the *MECP2* gene, corresponding to p.Arg106Trp, which is known to be associated with a severe phenotype (Figure 2A,B).

Supplementary Table S2 shows the cohort’s clinical characteristics and mutation landscape.

### 3.2. RTT and Control Brain Histology

**Figure 3:**
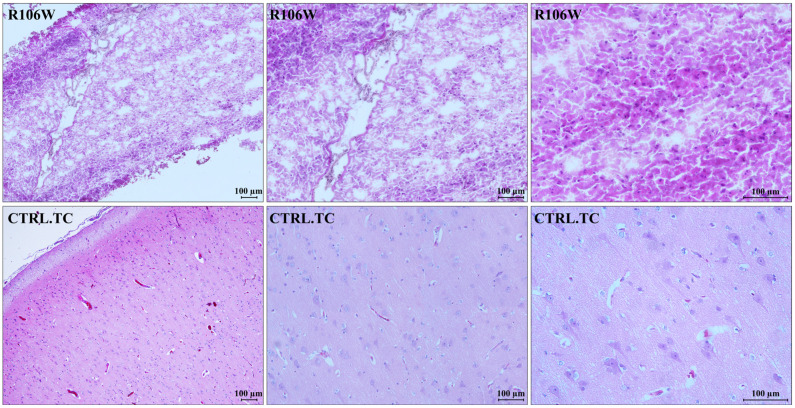
Histological characterization of brain specimens. A representative image of the RTT brain (R106W) temporal cortex, along with magnifications of specific areas, shows severe processing artifacts. The RTT brain tissue is partially hypocellular, with the presence of glial cells compatible with reactive gliosis (**upper panels**)**.** A representative image of the control brain temporal cortex (CTRL.TC), along with magnifications of specific areas, shows a fully represented neuronal population with normal architecture (**lower panels**). Counterstain: Hematoxylin and Eosin (H&E).

Histological analysis of the RTT brain specimen showed severe processing artifacts, likely due to poor tissue conservation. The temporal cortex of the RTT brain resulted in partial hypocellularity, with an increased glial cell component consistent with reactive gliosis. As already observed, neurons were absent, and consequently, grey matter was strongly reduced. In contrast, the control brain temporal cortex showed a fully represented neuronal population with normal architecture, which was lost in the RTT brain (Figure 3).

### 3.3. Skewed XCI Pattern in RTT Brain

Methylation studies by PCR and digestion with methylation-sensitive enzymes of the androgen receptor (AR) gene’s polymorphic allele allow for discrimination between the active and inactive X chromosomes.

**Figure 4:**
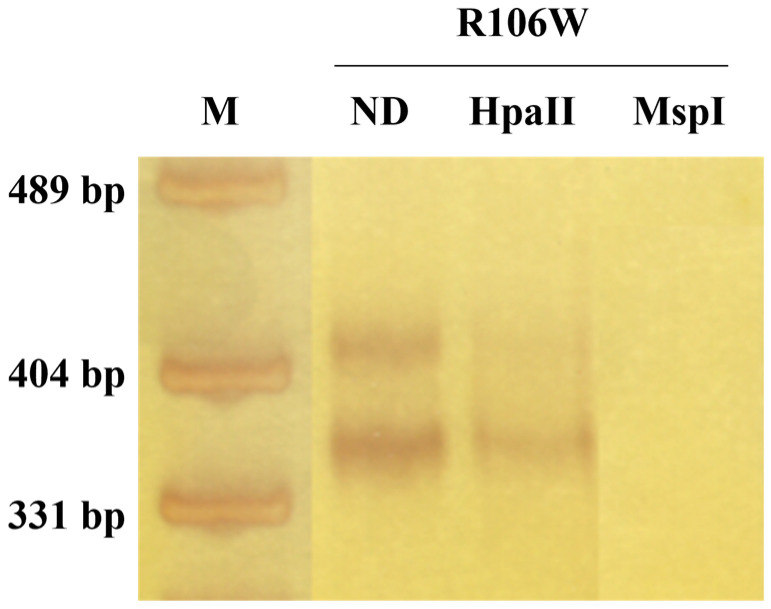
Skewed XCI pattern in RTT brain. XCI analysis of temporal cortex DNA from the RTT patient revealed extreme skewing (95:5 ratio). The fragments’ molecular weights are approximately 420 and 390 bp. ND: not digested. M: pUC19 DNA/MspI (HpaII) Marker (Thermo Fisher Scientific, Waltham, MA, USA).

XCI analysis of temporal cortex-derived DNA revealed extreme skewing (95:5 ratio) in the RTT patient, compared to the random XCI pattern observed in the control temporal cortex (Figure 4; Supplementary Figure S2).

### 3.4. MECP2 Transcript Analysis in RTT Brain Reveals Preferential Expression of Mutated Allele

**Figure 5:**
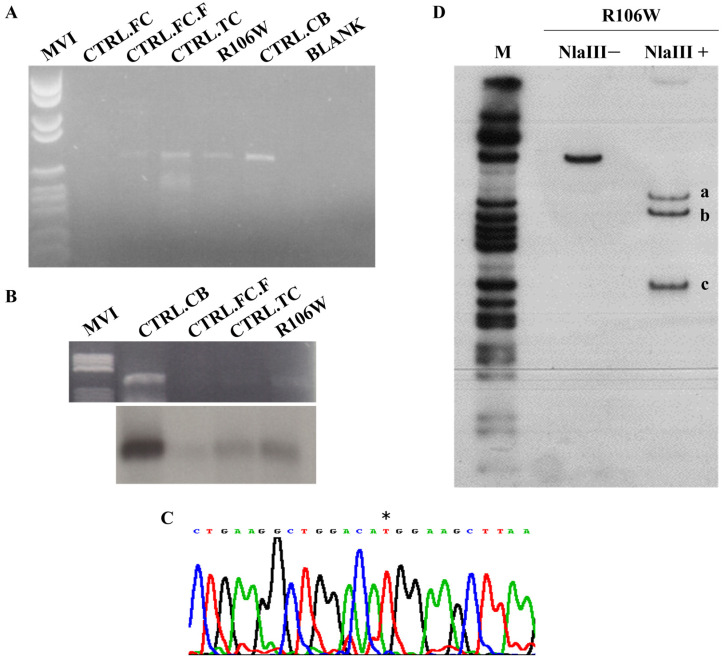
MECP2 transcriptional studies in the RTT brain carrying p.Arg106Trp mutation. (**A**) Gene-specific RT-PCR in the RTT brain temporal cortex (R106W) and four control tissues (two frontal cortices (CTRL.FC-CTRL.FC.F), temporal cortex (CTRL.TC), and cerebellum (CTRL.CB)). The *MECP2* transcript is clearly represented in 4 out of 5 tissues analyzed, including the RTT brain. (**B**) RT-PCR products were blotted and hybridized with an exon 4 *MECP2* probe. (**C**) RTT PCR product sequencing identified only the mutated *MECP2* transcript (*). (**D**). Hot-stop RT-PCR radiolabeled product was digested with the NlaIII restriction enzyme. Undigested transcript (NlaIII-) size: 410 bp; digested transcript (NlaIII+) sizes: 278 bp (a) and 132 bp (c) for the wild-type allele and 243 bp (b), 132 bp (c), and 35 bp (not detectable) for the mutated R106W allele. The R106W mutation creates a novel NlaIII restriction site. M: pBR322 *Hae III* Digest DNA Marker (Sigma-Aldrich, St. Louis, MO, USA).

We analyzed the RTT brain temporal cortex and four control tissues (temporal cortex—CTRL.TC; cerebellum—CTRL.CB; and two frontal cortices—CTRL.FC and CTRL.FC.F) by RT-PCR. Figure 5A shows that this initial qualitative analysis confirmed the presence of the *MECP2* transcript in four out of five tissues, including the RTT brain. Blotting and hybridization with an exon 4 *MECP2* probe of the RT-PCR products (Figure 5B) served as a secondary qualitative verification of transcript representation.

Sequence analysis by direct sequencing of the RTT brain RT-PCR product revealed the presence of the mutated *MECP2* transcript only, with the wild-type allele possibly being barely visible in the electropherogram (Figure 5C). While RT-PCR is not quantitative, this pattern suggests potential allelic imbalance.

Hot-stop RT-PCR with NlaIII digestion (Figure 5D) provided semi-quantitative validation, revealing a 10:1 ratio of mutant (R106W) to wild-type transcripts (1.89 ± 0.19, mean ± SEM, n = 3 replicates; Supplementary Figure S3).

### 3.5. MeCP2 Protein Expression in RTT Brain

**Figure 6:**
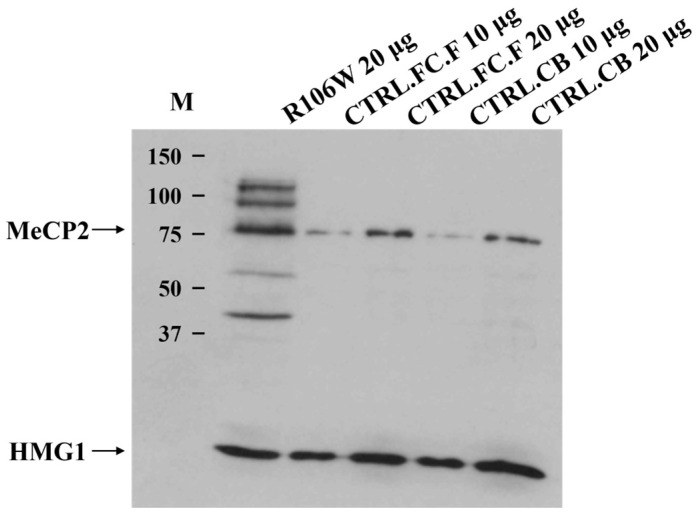
MeCP2 protein analysis in the RTT brain carrying p.Arg106Trp mutation. Western blot shows the presence of a protein of 75 kDa in RTT index case (R106W), control frontal cortex (CTRL.FC.F), and control cerebellum (CTRL.CB) (Anti-MeCP2 Antibody). Higher and lower fragments are well visible in RTT brain. Proteins were visualized using the ECL+ system (Amersham). MeCP2 protein levels (75 kDa band) were quantified by densitometric analysis using the AlphaImager™ Gel Imaging System (Alpha Innotech) and its integrated quantification software. M: Precision Plus Protein Standards MW (Bio-Rad, Hercules, CA, USA).

Western blot analysis of the RTT brain revealed the presence of a normally sized protein of 75 kDa, obviously not affected by the presence of the p.Arg106Trp (R106W) missense mutation. However, quantification using AlphaImager™ software revealed a 5.2 ± 0.8-fold increase (mean ± SEM, n = 3) in MeCP2 protein levels in the RTT brain compared to controls after normalization to HMG1 loading control (*p* = 0.02, Student’s *t*-test) (Figure 6).

Additionally, other higher and lower molecular weight protein fragments were visible only in the RTT brain, although their meaning remains uncertain. The loading control protein HMG1 was clearly detectable, supporting the high quality of both the Western blot experiment and the brain samples analyzed.

### 3.6. Spatial Localization of MECP2 Transcript in Human Control Brain Cerebellum and Temporal Cortex

**Figure 7:**
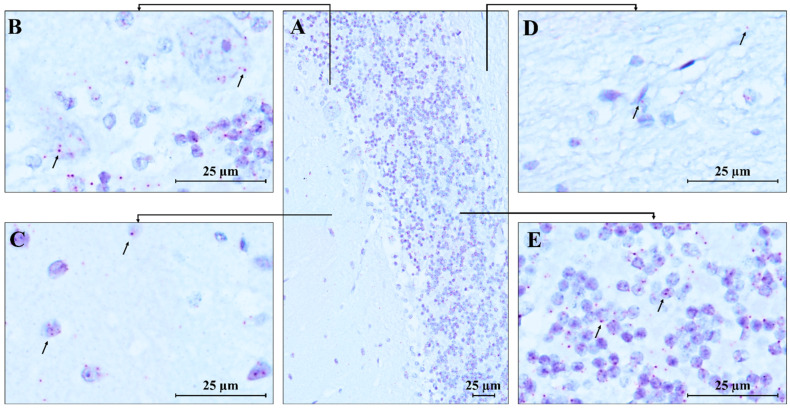
RNAscope ISH in control human cerebellum. A 20 ZZ probe targeting nucleotides between 355 and 1417 of the *MECP2* transcript was used. (**A**) Representative image of control human cerebellum (CTRL.CB); (**B**–**E**) Magnification of specific areas within the cerebellum. Each single *MECP2* transcript is represented as a distinct red dot (black arrows) in Purkinje cells (**B**), in neurons of the molecular layer (**C**), and the granule cell layer (**E**). Weaker signals are detected in the white matter (**D**). Counterstain: Gill’s Hematoxylin.

RNAscope ISH analysis revealed the spatial compartmentalization of the *MECP2* transcript in the normal human brain, specifically in the cerebellum (CTRL.CB) and temporal cortex (CTRL.TC). Hybridization signals were observed in the cerebellum, with strong expression in the Purkinje, molecular, and granular layers, while weaker signals were detected in the white matter, likely due to the lower density of neurons in this region (Figure 7). *MECP2* signals were predominantly localized within the cell bodies, with only a few red dots detected in the neuropil of the molecular layer, suggesting a highly specific and localized distribution of the transcript within the cells.

**Figure 8:**
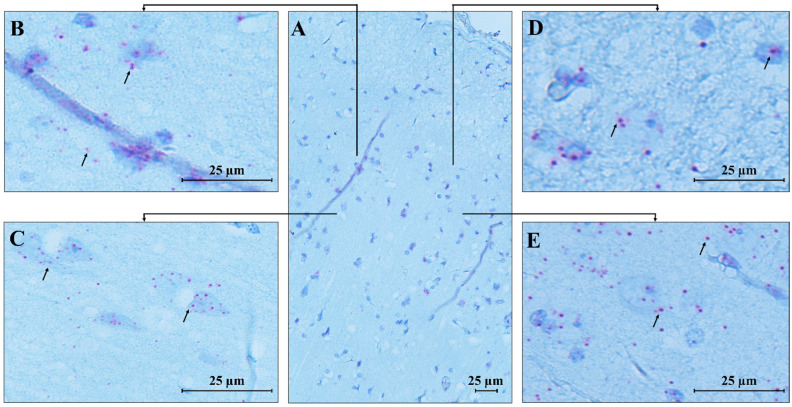
RNAscope ISH in control human temporal cortex. A 20 ZZ probe targeting nucleotides between 355 and 1417 of the *MECP2* transcript was used. (**A**) Representative image of control human temporal cortex (CTRL.TC); (**B**–**E**) Magnification of specific areas within the temporal cortex. Each single *MECP2* transcript is represented as a distinct red dot (black arrows) in cortical neurons and glial cells. Counterstain: Gill’s Hematoxylin.

An identical pattern of *MECP2* transcript expression was found in the temporal cortex (Figure 8), where high expression was observed in pyramidal neurons and granular cells. Low signals were detected in the molecular layer, which primarily consists of axons and dendrites, explaining the less pronounced expression in this area compared to the more densely populated cortical regions with cell bodies.

Furthermore, we detected the expression of the *MECP2* transcript in endothelial cells of blood vessels in both analyzed brain areas, supporting the impaired vascular function previously reported in RTT mouse models [15].

## 4. Discussion

MeCP2 is a protein crucial for neuronal development and epigenetic regulation since it binds to gene/genomic methylated regions, acting as a critical transcriptional regulator in the brain. It undergoes several post-translational modifications, including ubiquitination and sumoylation, which play important roles in gene regulation, synaptic plasticity, polymeric organization, and proteasomal degradation. For example, ubiquitin-mediated proteasomal degradation is crucial for maintaining MeCP2 homeostasis, thus supporting its silencing function on the brain transcriptome [16]. Thus, MeCP2 binds extensively to heterochromatin, acting as a chromatin remodeling factor, and the adaptive structure also favors post-translational modifications, adding a further layer of functional complexity to MeCP2 activity. Like other CpG-binding proteins, MeCP2’s function links methylation and histone deacetylation to a common repressive pathway, which culminates in closed chromatin conformation, mediating gene transcription repression [17].

Although the adoption of omics strategies has advanced our understanding of RTT pathogenesis, the molecular mechanisms underlying impaired brain development in RTT patients remain incompletely characterized. Recent studies in mouse models demonstrated that *Mecp2* mutations caused widespread bidirectional transcriptional dysregulation in the brain, with detailed hippocampal analyses revealing an early decrease in histone H3 acetylation at downregulated genes [7]. This observation suggests that MeCP2 loss of function or haploinsufficiency triggers a cascade of events that become “disease-independent”, leading to irreversible brain developmental dysfunction. This may explain the classical and rapid deterioration of cognitive function in RTT girls. Despite the extreme rarity of available RTT brain tissue for research, we had the opportunity to obtain and analyze a post-mortem brain sample from an RTT patient with the p.Arg106Trp missense mutation. Although tissue preservation was suboptimal (a frequent challenge with such rare specimens), we were able to conduct comprehensive histological examination, transcript/protein analyses, and XCI profiling. Our findings, consistent with prior reports on compromised neuropathological material [9], included reduced gray matter density, abnormal myelination patterns, and disorganized cortical architecture in the temporal cortex.

### 4.1. MECP2 Transcript Characterization and Protein Expression in RTT Brain

A previous report evaluating *MECP2* transcript levels in RTT brains (with A201V, T158M, and R255X mutations, distinct from our R106W case) found significantly lower transcript levels compared to controls [18]. While our conventional gene-specific RT-PCR confirmed the presence of the *MECP2* transcript across brain tissues, the RTT brain tissue unexpectedly revealed a prominent representation of the mutated allele. This finding was consistent with the extremely skewed XCI pattern observed in brain-extracted DNA and supported by semi-quantitative allele-specific assessment, showing that approximately 90% of *MECP2* transcripts originated from the mutated allele. These results suggest that while total *MECP2* transcript levels may be reduced in RTT (as reported for other mutations), our R106W case demonstrates a unique pattern of mutant allele predominance that warrants further investigation.

Western blot analysis, which cannot obviously discriminate between mutant and wild-type protein, showed markedly elevated MeCP2 protein levels compared to controls, a finding consistent with previous observations of efficient translation from mutant *Mecp2* transcripts in mouse models [19]. A possible explanation could be the increased resistance to degradation and the more complex homeostasis of the mutated protein [18].

The combination of elevated MeCP2 levels, allele-specific transcription results, and skewed XCI pattern points to a severe loss-of-function mechanism, rather than mere haploinsufficiency, as the likely cause of the RTT phenotype in this patient. The predominance of the mutated transcript suggests that RNA-targeting strategies could effectively mitigate dominant-negative effects in RTT patients with similar molecular profiles.

Notably, control brain regions (including the cerebellum) also exhibited substantial MeCP2 protein levels, indicating that absolute protein quantity alone cannot explain the pathological effects. It is known that the MeCP2 protein undergoes post-translational modifications, such as ubiquitination and sumoylation, which are related to its multifunctional epigenetic roles [20,21].

Notably, the R106W mutation (associated with severe clinical phenotypes) could exacerbate these phenomena. This substitution replaces arginine-106 in the MBD with a bulkier, hydrophobic tryptophan, inducing significant structural destabilization. The mutation disrupts hydrogen bonds with key residues (M94, D156, T158, V159), distorts local structure, and impairs water-mediated DNA recognition (e.g., via Tyr-123) and interdomain allostery [22]. Circular dichroism spectroscopy further reveals that R106W reduces α-helical content while increasing β-sheet formation [23,24], and unlike wild-type MeCP2, the mutant fails to undergo DNA-binding-induced conformational changes. Given this pronounced structural perturbation, we hypothesize that the additional bands observed in RTT brain Western blots may arise from enhanced post-translational processing and/or fragmentation of the mutant protein. The R106W variant’s misfolded or partially unfolded state could expose protease-sensitive sites that are normally shielded in the wild-type protein, rendering it more susceptible to cleavage. Alternatively, the mutation might dysregulate modification pathways (e.g., phosphorylation, sumoylation), leading to aberrant isoform accumulation. This hypothesis aligns with the banding patterns seen in proliferating cells [25] (where MeCP2 processing may differ from post-mitotic neurons) and could explain the distinctive profile in R106W-derived tissues. Further studies should investigate whether R106W’s structural instability directly increases its proteolytic sensitivity or alters its post-translational modification landscape, potentially contributing to RTT pathophysiology.

### 4.2. MECP2 Transcript Localization in Human Control Brain Regions Supports MeCP2’s Role in Neuronal Function

For the first time, we used a *MECP2* RNAscope ISH assay to study control brain tissues (the RTT brain tissue was unsuitable for this method due to its poor conservation). The *MECP2* transcript was abundant in the temporal cortex and cerebellum areas, with some area- or cell-specific distribution. *MECP2* mRNA was localized mainly in the intracellular compartment, consistent with its regulatory function, which requires efficient and continuous nuclear protein import to mediate DNA CpG binding activity. The *MECP2* transcript was also abundant in nuclei, likely reflecting a high transcription rate. In the cerebellum, Purkinje cells were enriched in *MECP2* mRNA dots, supporting the high MeCP2 expression. This high MeCP2 expression is important for maintaining normal neuronal function, particularly in Purkinje cells, which are essential for coordinating motor movements and also orchestrating connections in the cerebellar-cortical tract [26]. This finding is fully in line with previously published data showing that MeCP2 deficiency in Purkinje cells reduces their intrinsic excitability through a signaling pathway involving the small-conductance calcium-activated potassium channel (PTP1B) and the receptor for brain-derived neurotrophic factor (TrkB). Disruption of this pathway leads to autistic-like behaviors and vestibulo-cerebellar motor learning impairments observed in patients with RTT [27]. Besides its role in the cerebellum, MeCP2 expression in the temporal cortex is known to be responsible for maintaining normal neuronal function and synaptic plasticity, both of which are crucial for cognitive and emotional processes [28]. Therefore, its loss leads to the broad spectrum of behavioral, cognitive, and sensory dysfunction seen in RTT. In conclusion, through RNAscope studies, we demonstrated abundant expression of *MECP2* transcript in the studied brain areas, with a previously unreported prominent nuclear localization, highlighting the nuclear-related MeCP2 functions as well as in cerebellar circuits.

### 4.3. MECP2-Linked XCI and L1 Retrotransposition in RTT Brain

Although this represents a single case, we provide comprehensive molecular profiling of *MECP2* in RTT brain tissue, including allele-specific transcript quantification and XCI pattern. Our data showed skewed XCI favoring the inactivation of the *MECP2* wild-type allele, resulting in the predominant expression of the mutated transcript. The underlying mechanism responsible for this preferential inactivation remains unknown.

As previously described, the XCI pattern observed in the RTT brain is predominantly balanced, with intra-individual variability across different neuroanatomical regions [29]. Gibson et al. characterized the XCI pattern in different regions of RTT brains carrying different mutations, including R106W, and found that in the temporal cortex of an RTT patient with the R106W mutation, the XCI pattern was closer to the skewing threshold, which aligns with our findings [30]. Similarly, Xiol et al. investigated a nonsense mutation located in the TRD domain across several brain areas, reporting that in some regions, the XCI pattern was closer to the skewing threshold [31].

Nevertheless, we propose an intriguing hypothesis to explain the skewed XCI pattern observed in different brain areas. This hypothesis links the mutated *MECP2* gene to the X-chromosome-imprinting process.

It is well known that XCI is the silencing mechanism adopted by eutherian mammals to equalize the expression of X-linked genes between females and males early in embryonic development [32]. Only genes located in pseudo-autosomal regions, such as PAR1 (Xp22) and PAR2 (Xq28), are exempt from the XCI mechanism [33]. A complex non-coding RNA (ncRNA)-mediated mechanism regulates XCI. The master ncRNA, which primes this process, is the *XIST* transcript, which maps to the X-inactivation center (Xq13) and is required *in cis* for the initiation and propagation of silencing. Other cis/trans factors also orchestrate the whole process [34]. *XIST* is highly expressed by the inactive X chromosome only [35], and its function is to recruit both silencing RNAs and proteins to form a large “coating” complex that labels the inactive X chromosome. Increased *XIST* expression represents the key initiation signal driving XCI, highlighting the central role of this ncRNA. XIST spreads along the X chromosome more efficiently than on autosomes due to the presence of “way stations” or “boosters”, which consist of interspersed LINE-1 elements (L1s). Comparative genomic analyses from the Human Genome Project have shown that X chromosomes in humans and mice are about 26% enriched in L1s, compared to only 13% in autosome DNA [36]. L1s are retroelements interspersed in mammalian genomes, most of which are inactive or weakly and sporadically active at the somatic level in Homo sapiens and other mammals. This means that L1s are not routinely retrotranscribed and, therefore, do not typically retrotranspose and insert into genomic sequences [37]. Studies on rodents and neuronal-induced RTT pluripotent stem cells have shown that mutated *MECP2* cells exhibit an increased propensity for L1 retrotransposition, suggesting a role for *MECP2*-mediated DNA methylation in regulating this process.

Indeed, MeCP2 activity in controlling L1 mobility in neurons has been well-documented [38]. More recently, whole genome sequencing has shown that L1 retrotransposition is generally increased in neurodevelopmental disorders, including RTT [39]. Therefore, a link between *MECP2* and L1 elements seems evident and quite attractive as a model, with L1s serving as boosters that enable *XIST* to spread the inactivation signal along the X chromosome. In an intriguing hypothesis, we may speculate that *MECP2* may have a cooperative role in spreading *XIST* onto L1 boosters via L1 retrotranscription regulation. If this mechanism is operative, mutated MeCP2 proteins could promote inactivation of the X chromosome carrying the wild-type *MECP2* allele (“in trans”) through enhanced L1 retrotransposition activity. This could affect *MECP2* transcription and may account for the increased transcription and expression of the mutated allele, leading to a profound deficiency in wild-type protein and a loss of the function mechanism. This hypothesis may deserve further investigation.

### 4.4. MECP2 Mutation Landscape and RTT Phenotypic Characterization

As previously proposed, the spontaneous deamination of methylated cytosine at CpG dinucleotides seems to be the most probable mutational mechanism for RTT patients, giving rise to the high mutation rate and the fact that almost all *MECP2* mutations are de novo [40]. The occurrence of point mutations at CpG hotspots is over 50%. Mutations that do not preserve the function of the MBD and TRD domains are associated with a more severe phenotype, underlying the importance of keeping these two domains intact. However, considering the mutation landscape, other MeCP2 motifs and domains are also important for MeCP2 functions. Terminal deletions occur close to poly-histidine and polyproline stretches, which share homology of 35% identity and 50% positivity in a 75 amino acid region with two brain-specific factors: brain-specific factor 1 (BF-1) and forkhead 4 (FKH4), members of the forkhead gene family. Both factors are expressed exclusively in neurons of the developing telencephalon [41]. Homology is also observed with the retroviral oncogene *qin*, which binds the same consensus sequence of BF-1 and acts as a transcriptional repressor [42]. In cases where truncated proteins occur, a haploinsufficiency/loss of function effect might not be excluded.

In our RTT female cohort, 44 patients had classical RTT, and 10 had atypical RTT.

Among the classical RTT patients, the age at diagnosis was 1 year 4 months to 50 years. The majority of identified mutations were nonsense mutations (18 cases) and missense mutations (14 cases). Among the nonsense mutations, the most frequent were p.Arg168Ter (six cases), p.Arg255Ter (five cases), and p.Arg270Ter (three cases). Frameshift mutations were found in eight cases, with the most frequent being p.Gly269AlafsTer20 and p.Leu386HisfsTer5 (two cases each). Among the missense mutations, the most frequent were p.Thr158Met (six cases), p.Arg306Cys, and p.Pro152Arg (two cases each). Among the deletions, there were two cases involving exons 3–4 and two cases involving exon 4-3′UTR.

Among the atypical RTT patients, the age at diagnosis ranged from 2 years to 18 years 3 months. The mutations identified in this group were, in order of frequency, three nonsense mutations (p.Arg270Ter, p.Arg294Ter, p.Arg168Ter), three deletions (two involving exons 3–4 and one involving exon 4-3′UTR), two N-terminal deletions (both involving exons 1–2), one missense mutation (p.Leu301Val), and one frameshift mutation (c.603_606dupGGCC).

The phenotype of male subjects (two cases) was characterized by global intellectual disability, which significantly differs from the typical RTT phenotype, as previously described.

In terms of mutation frequency and mutation landscape, our data align with those reported in the literature.

## 5. Conclusions

We report *MECP2* transcription and XCI profile in an RTT brain specimen. The data suggest that MeCP2 plays a role not only as a global brain transcriptional repressor but also as an actor in the XCI process, hypothetically, through the regulation of L1 retroelements. *MECP2* RNAscope analysis in control brain areas fully supports the nuclear role of MeCP2 and its cerebellum-related functions.

## Data Availability

All data presented in this paper are fully available.

## Supplementary Materials

The following supporting information can be downloaded at https://www.mdpi.com/article/10.3390/biom15050687/s1, Table S1: Table S1_ Oligonucleotide sequences; Table S2: Table S2_Cohort clinical characteristics and mutation landscape; Figure S1: Figure S1_XCI pattern analysis protocol; Figure S2: Figure S2_XCI pattern in the control brain temporal cortex; Figure S3: Figure S3_NlaIII restriction digestion analysis of Hot-stop RT-PCR products.

## Abbreviations

The following abbreviations are used in this manuscript:

*MECP2*	Methyl-CpG-binding protein 2 gene (human)
MeCP2	Methyl-CpG-binding protein 2 (human)
Mecp2	Methyl-CpG-binding protein 2 gene (mouse)
Mecp2	Methyl-CpG-binding protein 2 (mouse)
RTT	Rett syndrome
ID	Intellectual disability
CNS	Central Nervous System
MBD	Methyl-binding domain
TRD	Transcriptional repression domain
CTD	C-terminal domain
5mC	5-methylcytosine
HDAC3	Histone deacetylase 3
TBL1	Transducing beta-like protein 1
NCoR	Nuclear receptor co-repressor
SMRT	Silencing mediator of retinoic acid and thyroid hormone receptor
GPS2	G protein suppressor 2
CREB1	cAMP response element-binding protein
XCI	X-chromosome inactivation
ASDs	Autism spectrum disorders
ISH	In situ hybridization
MLPA	Multiplex ligation probe amplification
FFPE	Formalin-Fixed-Paraffin-Embedded
H&E	Hematoxylin and Eosin
OD	Optical Density
PPIB	Cyclophilin B
DapB	4-hydroxy-tetrahydrodipicolinate reductase
NTD	N-Terminal domain
AR	Androgen receptor
PTP1B	Small-conductance calcium-activated potassium channel
TrkB	Receptor for brain-derived neurotrophic factor
NcRNA	Non-coding RNA
L1s	LINE-1 elements
BF-1	Brain-specific factor-1
FKH4	Forkhead 4
