# Supplementary material for: MECP2 mRNA Profile in Brain Tissues from a Rett Syndrome Patient and Three Human Controls: Mutated Allele Preferential Transcription and In Situ RNA Mapping
Source: Biomolecules. 2025 May 8;15(5):687. doi: 10.3390/biom15050687 (PMC12108707; doi:10.3390/biom15050687)
Supplement: Supplementary file 1 [file biomolecules-15-00687-s001.zip › Figure S1_XCI pattern analysis protocol.pdf]

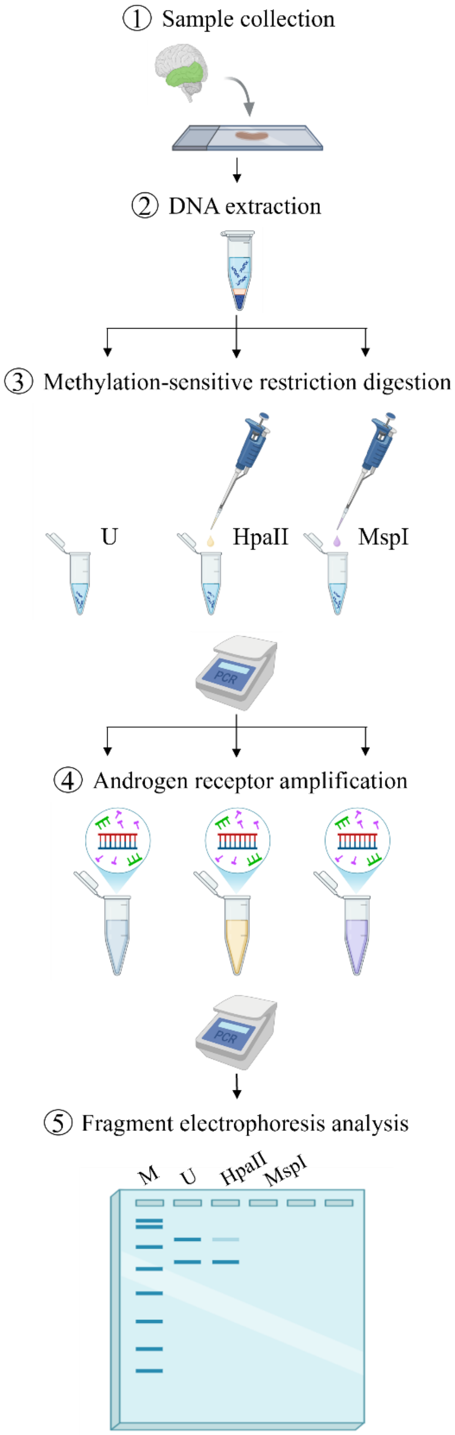

Figure S1. *X-chromosome inactivation (XCI) pattern analysis using HUMARA (Human Androgen Receptor Assay) and methylation-sensitive restriction enzymes*. The XCI analysis was performed using HUMARA assay on genomic DNA extracted from the temporal cortex tissue (**1-2**). Three parallel reactions were set up: the DNA was incubated without the restriction enzymes (U), with HpaII, and with MspI. The undigested control (U) was included to assess PCR efficiency and DNA integrity, while HpaII and MspI digestions allowed methylation-specific detection.

All samples were incubated for 16 hours at 37°C, followed by enzyme inactivation at 65°C for 20 minutes (**3**). Following digestion, androgen receptor amplification was performed using the following protocol: 94°C for 5 minutes, hold; 94°C for 45 seconds, 61°C for 45 seconds, 72°C for 1 minute, 35 cycles; 72°C for 5 minutes, hold (**4**). PCR products were separated by electrophoresis on 12% polyacrylamide gels and visualized using silver nitrate staining (**5**).
