# Supplementary material for: MECP2 mRNA Profile in Brain Tissues from a Rett Syndrome Patient and Three Human Controls: Mutated Allele Preferential Transcription and In Situ RNA Mapping
Source: Biomolecules. 2025 May 8;15(5):687. doi: 10.3390/biom15050687 (PMC12108707; doi:10.3390/biom15050687)
Supplement: Supplementary file 1 [file biomolecules-15-00687-s001.zip › Figure S2_XCI pattern in the control brain temporal cortex.pdf]

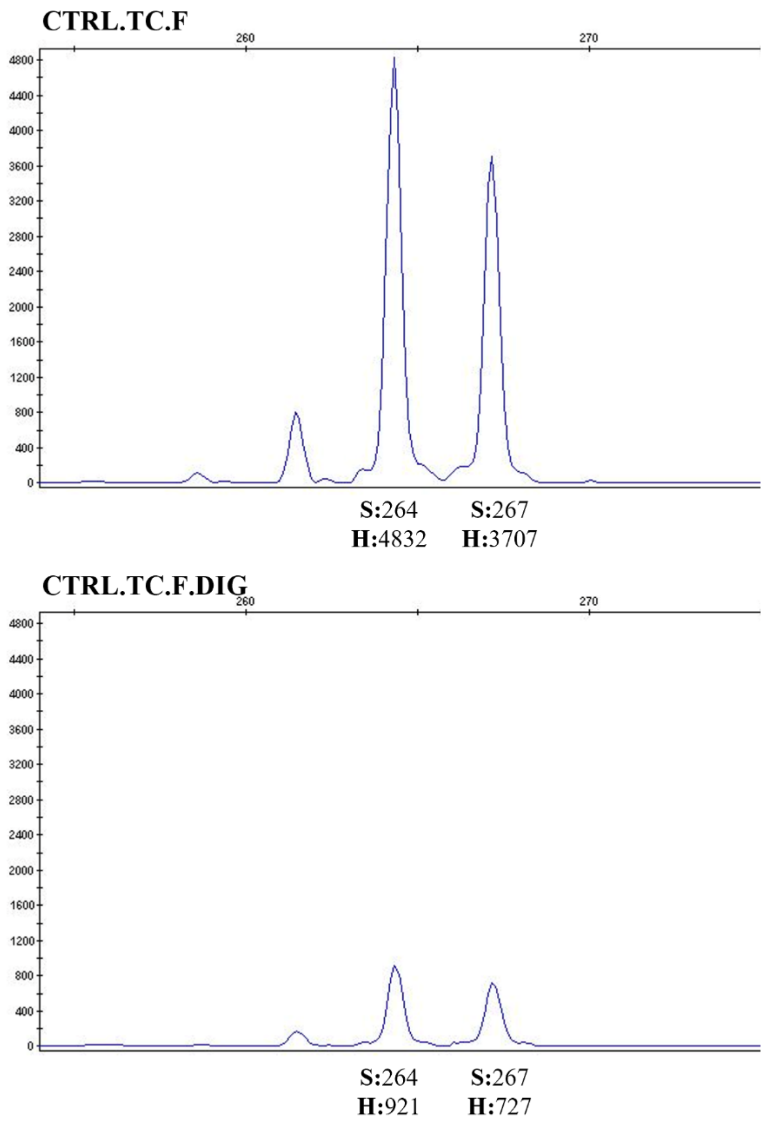

Figure S2. *Random XCI pattern in the control brain temporal cortex.* The XCI analysis was performed using HUMARA test on DNA extracted from the temporal cortex of the control brain (CTRL.TC.F). Fragments detection was carried out on the ABI PRISM 3500DX. The analysis revealed the presence of two fragments, respectively of 14 and 15 CAG repeats, consistent with a heterozygous genotype $(CAG)_{14}/(CAG)_{15}$. Following enzymatic digestion (CTRL.TC.F.DIG), the ratio between the two alleles was 49:51, consistent with a random XCI pattern. S: size; H: height.
