# Supplementary material for: MECP2 mRNA Profile in Brain Tissues from a Rett Syndrome Patient and Three Human Controls: Mutated Allele Preferential Transcription and In Situ RNA Mapping
Source: Biomolecules. 2025 May 8;15(5):687. doi: 10.3390/biom15050687 (PMC12108707; doi:10.3390/biom15050687)
Supplement: Supplementary file 1 [file biomolecules-15-00687-s001.zip › Figure S3_ NlaIII restriction digestion analysis of Hot-stop RT-PCR products.pdf]

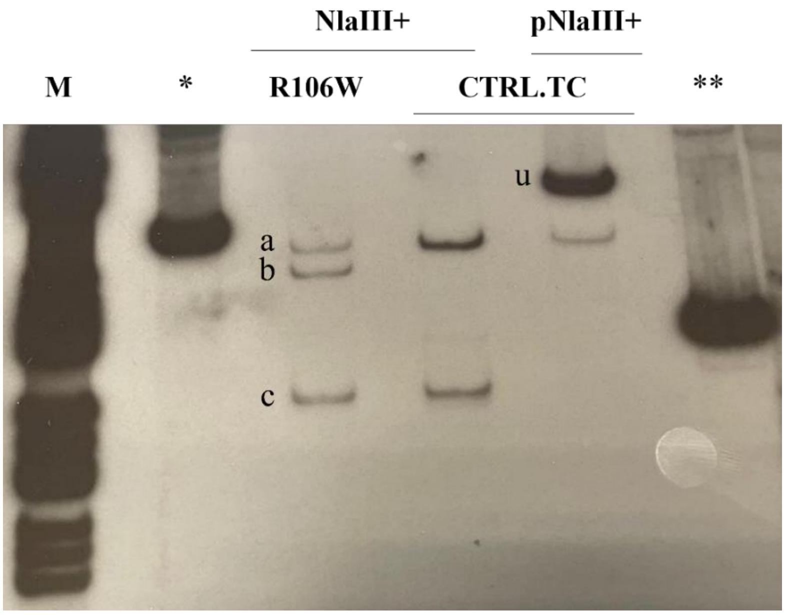

Figure S3. *NlaIII restriction digestion analysis of Hot-stop RT-PCR products.* Radiolabeled *MECP2* transcripts from RTT brain temporal cortex (R106W, mutant) and control temporal cortex (CTRL.TC, *wild-type*) were digested with NlaIII (2.5-hour incubation, NlaIII+). The R106W mutation introduces a novel NlaIII site, yielding allele-specific fragments:

***Wild-type* (CTRL.TC):** 278 bp (a) + 132 bp (c) (full digestion).

Mutant (R106W): 243 bp (b) + 132 bp (c) + 35 bp (not detectable).

Undigested (u): 410 bp (full-length transcript).

Controls and calibration: **pNlaIII+:** Partial digestion (25-minute incubation) of *wild-type* transcript, showing intermediate cleavage products. ***/**:** 300 bp and 177 bp radiolabeled reference fragments (that were included for size calibration during autoradiography optimization). **M:** pBR322 *HaeIII* Digest DNA Marker (Sigma-Aldrich).
