# Supplementary figures and images for: MECP2 mRNA Profile in Brain Tissues from a Rett Syndrome Patient and Three Human Controls: Mutated Allele Preferential Transcription and In Situ RNA Mapping
Source: Biomolecules. 2025 May 8;15(5):687. doi: 10.3390/biom15050687 (PMC12108707; doi:10.3390/biom15050687)

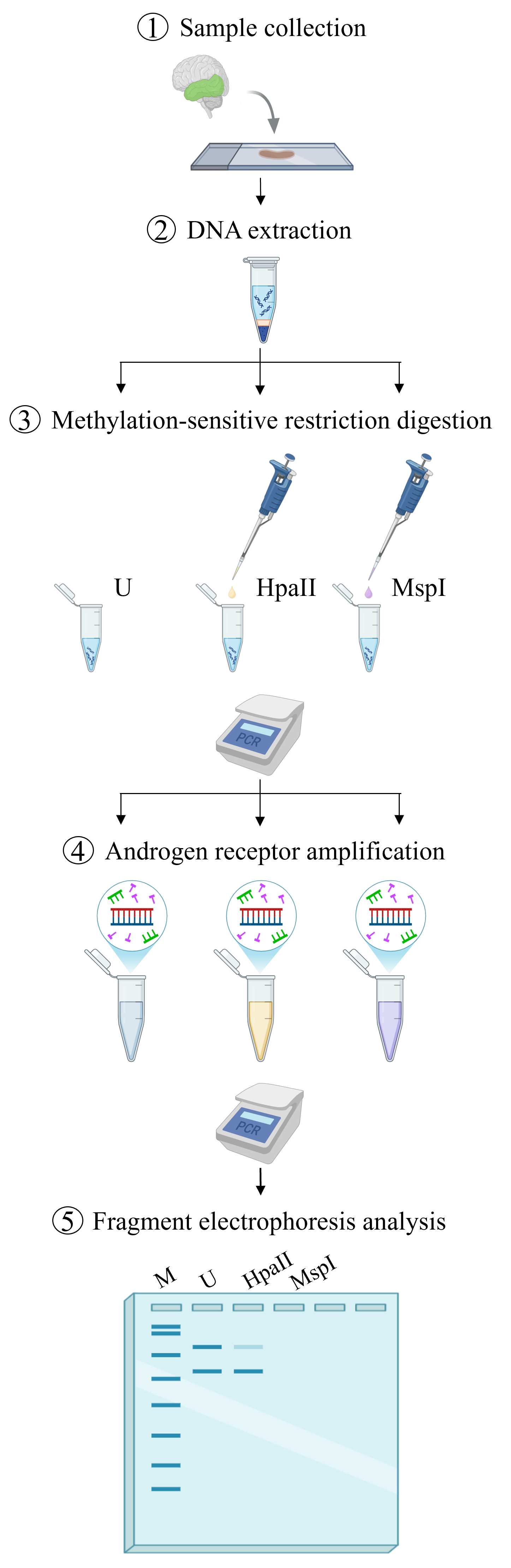

Supplement: Supplementary file 1 [file biomolecules-15-00687-s001.zip › Figure S1.tif]

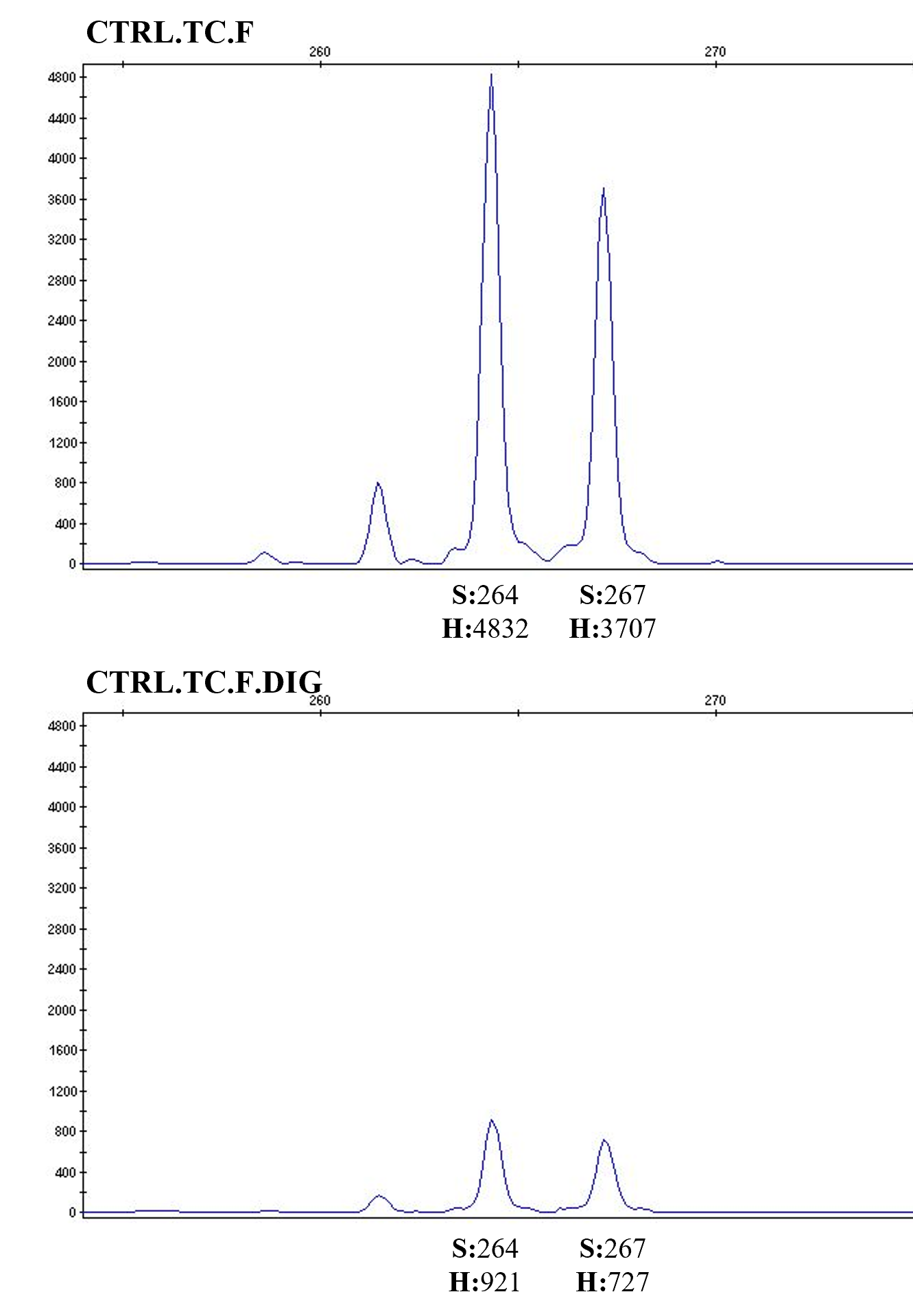

Supplement: Supplementary file 1 [file biomolecules-15-00687-s001.zip › Figure S2.tif]

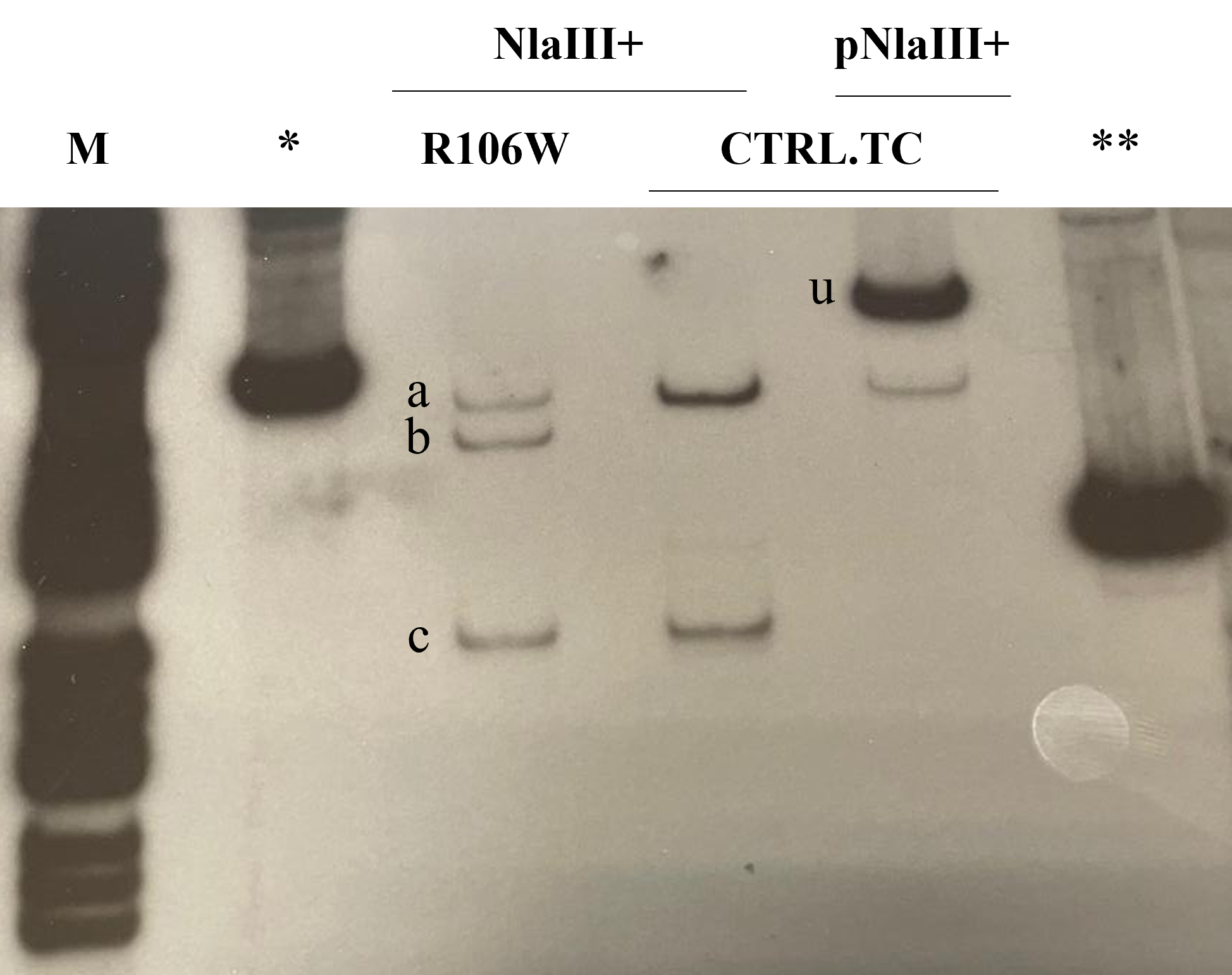

Supplement: Supplementary file 1 [file biomolecules-15-00687-s001.zip › Figure S3.tif]
